# Supplementary material for: Appetitive Motivation and Associated Neurobiology Change Differentially across the Life Course of Mouse Offspring Exposed to Peri- and Postnatal High Fat Feeding
Source: Nutrients. 2022 Dec 4;14(23):5161. doi: 10.3390/nu14235161 (PMC9735866; doi:10.3390/nu14235161)
Supplement: Supplementary file 1 [file nutrients-14-05161-s001.zip › Supplemental Figues Legend.pdf]

Supplemental Figure Legend

Supplemental Figure S1. Behavioural task outputs of 6-month-old male (A, D, G, J) and female (B, E, H, K) offspring and comparison between males and females (C, F, I, L) during PR2 testing. These include latency to collect reward (A-C), number of magazine entries/second (D-F), number of front beam breaks/second (G-I) and number of rear beam breaks/second (J-L). *$p<0.05$, **$p<0.01$, ***$p<0.001$, ****$p<0.0001$, one-way or two-way ANOVA with Sidak's post hoc.

Supplemental Figure S2. Behavioural task outputs of 12-month-old male (A, D, G, J) and female (B, E, H, K) offspring and comparison between males and females (C, F, I, L) on latency to collect reward (A-C), number of magazine entries/second (D-F), number of front beam breaks/second (G-I) and number of rear beam breaks/second (J-L) during PR2 testing. **$p<0.01$, ***$p<0.001$, one-way or two-way ANOVA with Sidak's post hoc.

Supplemental Figure S3. Comparison of latency to collect reward (A and B), number of magazine entries/second (C and D), number of front beam breaks/second (E and F) and number of rear beam breaks/second (G and H) in male (A, C, E, G) and female (B, D, F, H) offspring tested on PR2 at 6- vs 12-months of age. *$p<0.05$, **$p<0.01$, ***$p<0.001$, two-way, repeated measures ANOVA with Sidak's post hoc.

Supplemental Figure S4. Relative mRNA levels of *Drd1a* (A, D, G), *Drd2* (B, H), *Oprm1* (C, E, I) and *Crn1* (F, J) in the nucleus accumbens (NAc, A-C), caudate-putamen (CPu, E-F) and prefrontal cortex (PFC, G-J) of 10-month-old male and female offspring groups. For diet comparisons, mRNA levels are expressed relative to the male (M) or female (F) C/C group. For sex comparisons, levels are expressed relative to the males in each diet group. One-way or two-way ANOVA with Sidak's post hoc.

Supplemental Figure S5. Relative mRNA levels of *Drd2* (A, D), *Crn1* (B, F, H), *Drd1a* (C) and *Oprm1* (E, G) in the nucleus accumbens (NAc, A, B),), caudate-putamen (CPu, C-F) and prefrontal cortex (PFC, G, H) of 16-month-old male and female offspring groups. For diet comparisons, mRNA levels are expressed relative to the male (M) or female (F) C/C group. For sex comparisons, levels are expressed relative to the males in each diet group. *$p<0.05$, two-way ANOVA with Sidak's post hoc.

Supplemental Figure S6. Relative mRNA levels of *Crn1* (A, B), *Oprm1* (C, D, G, H) and *Drd1a* (E, F) in the nucleus accumbens (NAc, A-D), and prefrontal cortex (PFC, E-H) between 10- and 16-month-old male (A, C, E, G) and female (B, D, F, H) offspring groups. mRNA levels are expressed relative to 16-month-old animals. *$p<0.05$, two-way ANOVA with Sidak's post hoc.
