# Supplementary material for: Appetitive Motivation and Associated Neurobiology Change Differentially across the Life Course of Mouse Offspring Exposed to Peri- and Postnatal High Fat Feeding
Source: Nutrients. 2022 Dec 4;14(23):5161. doi: 10.3390/nu14235161 (PMC9735866; doi:10.3390/nu14235161)
Supplement: Supplementary file 1 [file nutrients-14-05161-s001.zip › Supplemental Table S1.pdf]

Supplemental Table S1. Primer sequences used in RT-qPCR analyses.

Gene	Forward primer	Reverse primer
Actb	GATGTATGAAGGCTTTGGTC	TGTGCACTTTTATTGGTCTC
Cnr1	AATGCCATTTAGGTGTTCTG	ATAGGTCTTAGAACCAACCC
Drd1a	ACTGACTCCAGTGTATTCTC	TTGGATACAGCAAACAACTC
Drd2	TTGTTCTTGGTGTGTTCATC	TATAGATGATGGGGTTCACG
Oprm1	ACTCATGTTGAAAAACCCTG	CTGTGTTCAGATGACATTCAC

Cycling parameters:

1. 95°C for 15 mins
2. 94°C for 15 sec
3. 54°C for 30 sec (*oprm1, Drd2, Crn1, b-actin)* or 52°C for 30 sec (*Drd1a*)
4. 72°C for 30 sec
5. Repeat steps 2-4 for 40 cycles
6. Hold at 4°C
