# Supplementary figures and images for: Appetitive Motivation and Associated Neurobiology Change Differentially across the Life Course of Mouse Offspring Exposed to Peri- and Postnatal High Fat Feeding
Source: Nutrients. 2022 Dec 4;14(23):5161. doi: 10.3390/nu14235161 (PMC9735866; doi:10.3390/nu14235161)

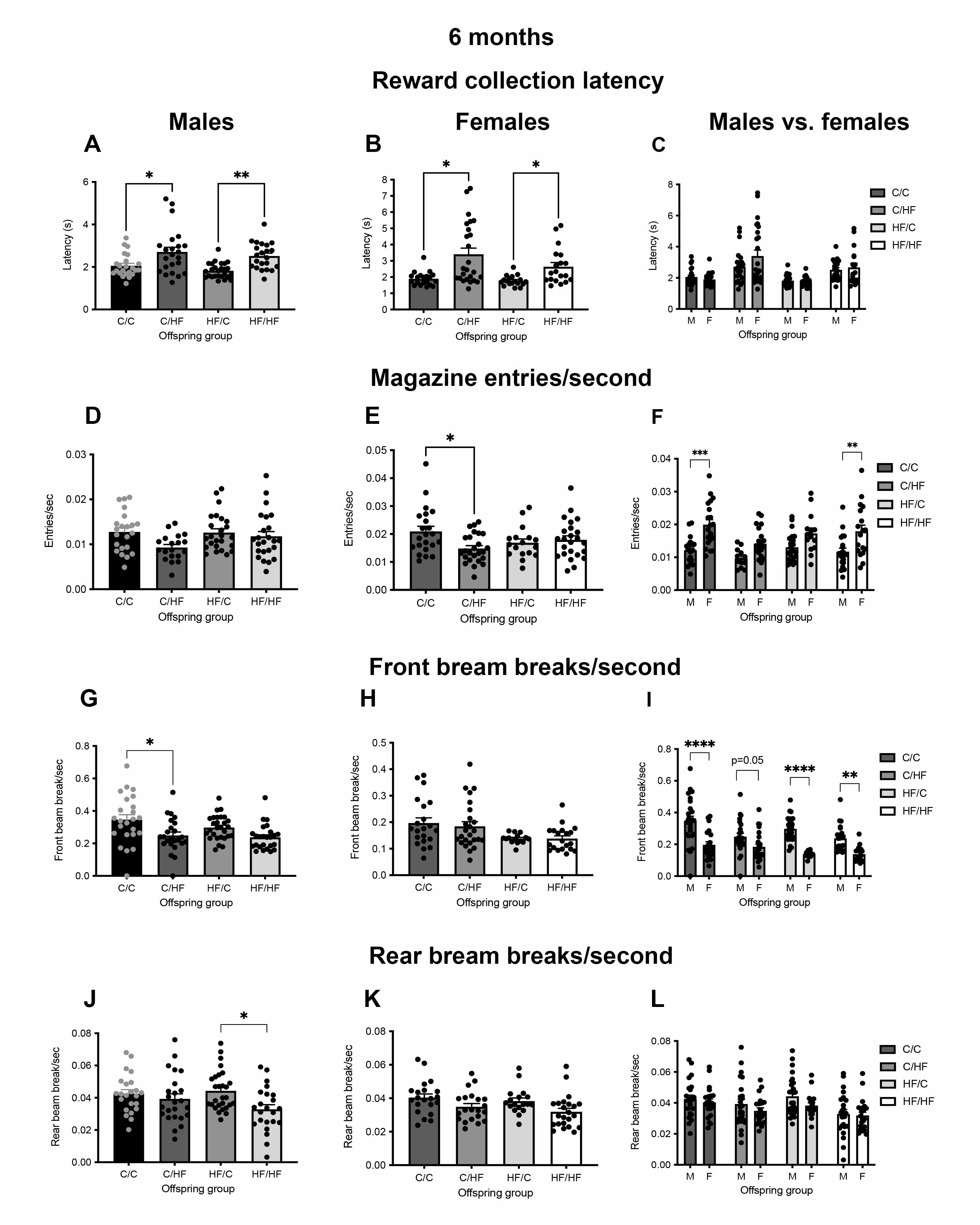

Supplement: Supplementary file 1 [file nutrients-14-05161-s001.zip › Supplemental Figure S1.jpg]

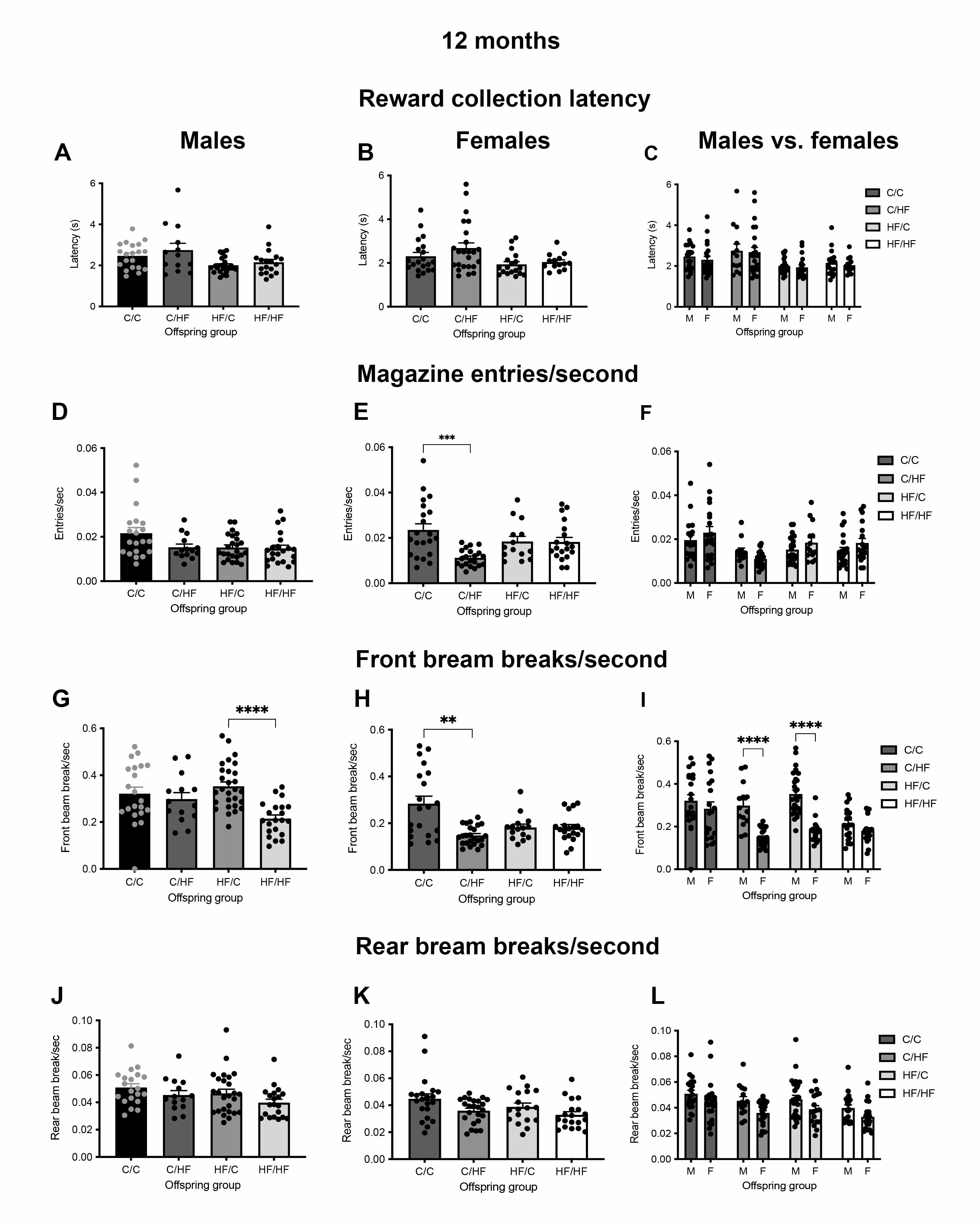

Supplement: Supplementary file 1 [file nutrients-14-05161-s001.zip › Supplemental Figure S2.jpg]

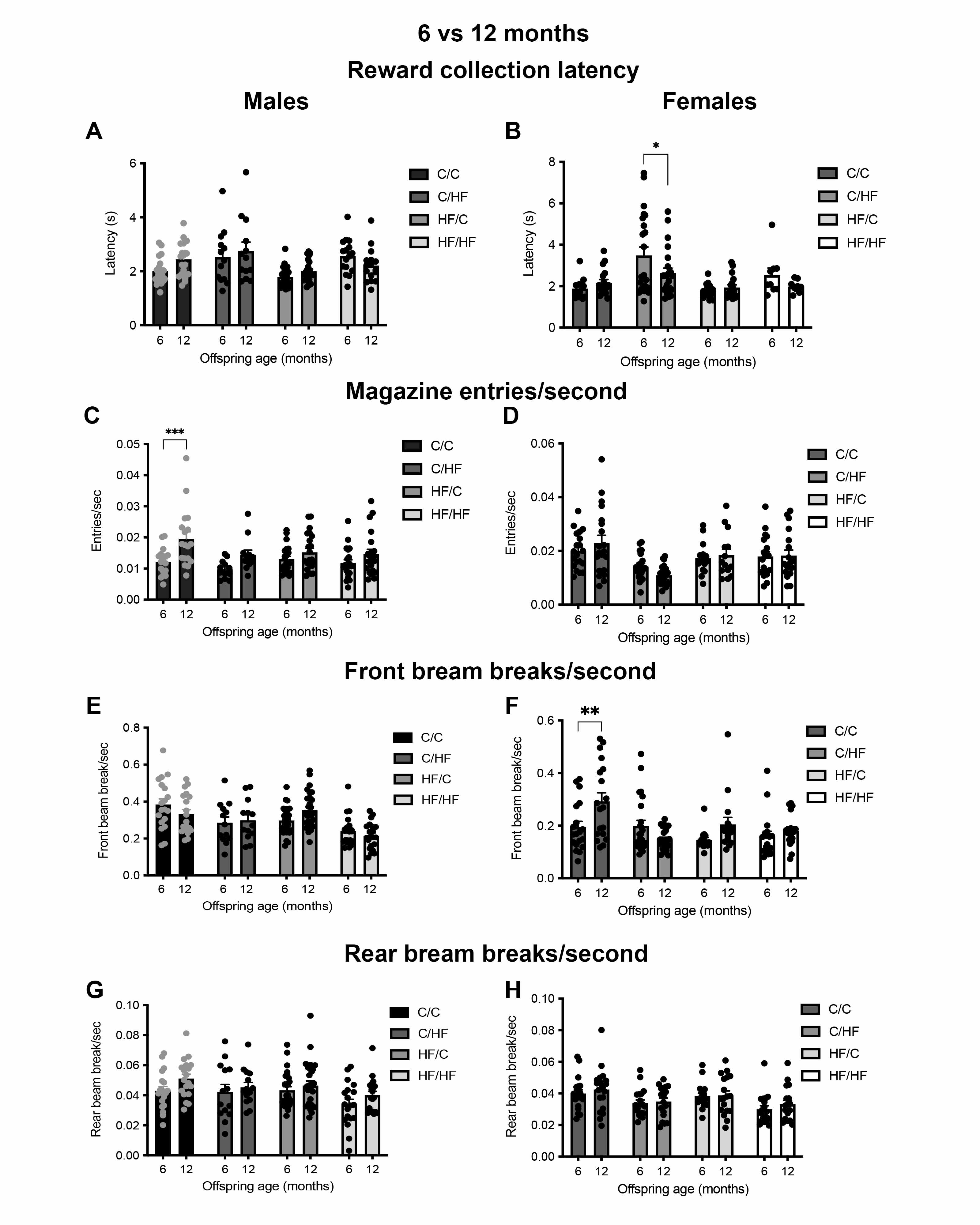

Supplement: Supplementary file 1 [file nutrients-14-05161-s001.zip › Supplemental Figure S3.jpg]

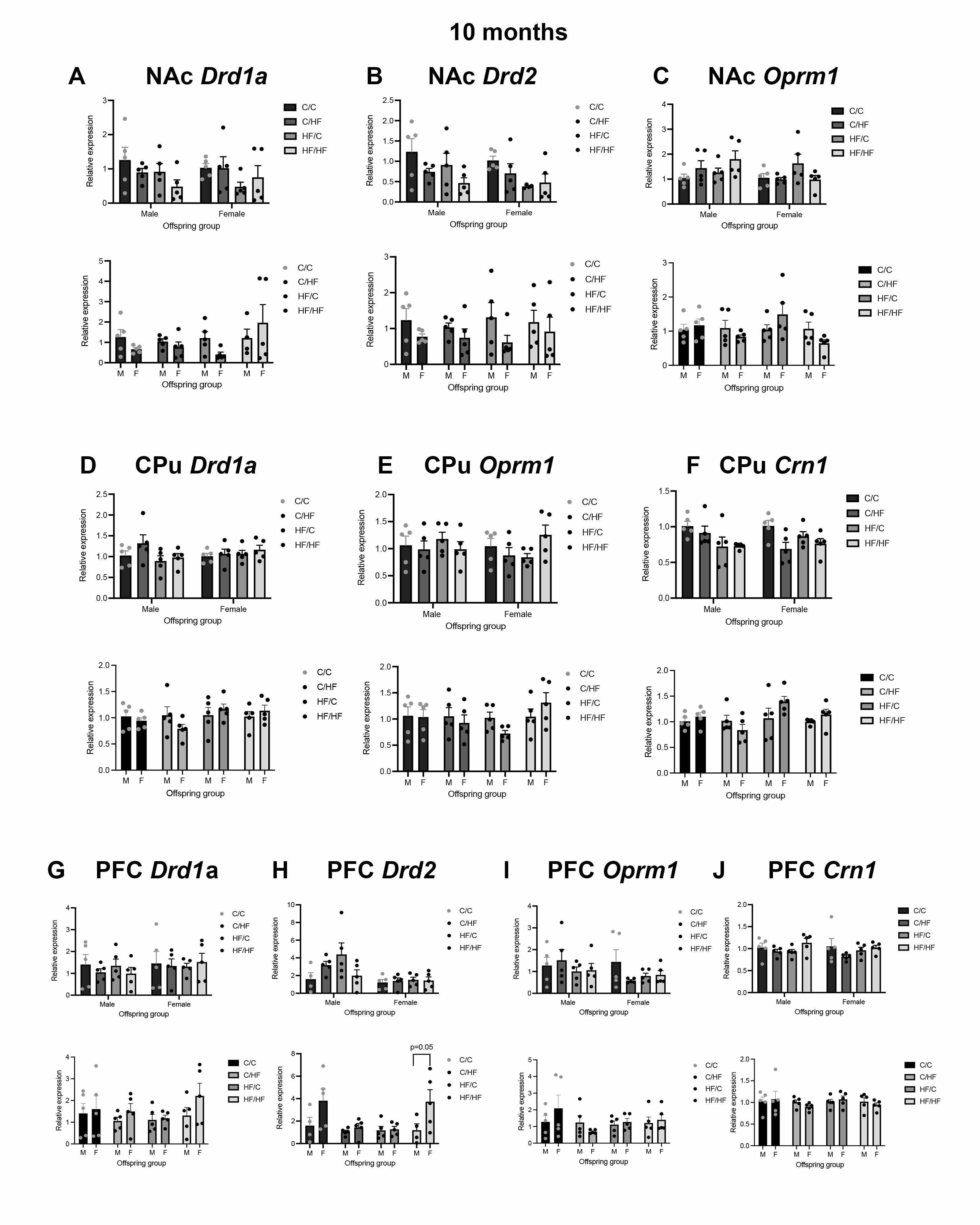

Supplement: Supplementary file 1 [file nutrients-14-05161-s001.zip › Supplemental Figure S4.jpg]

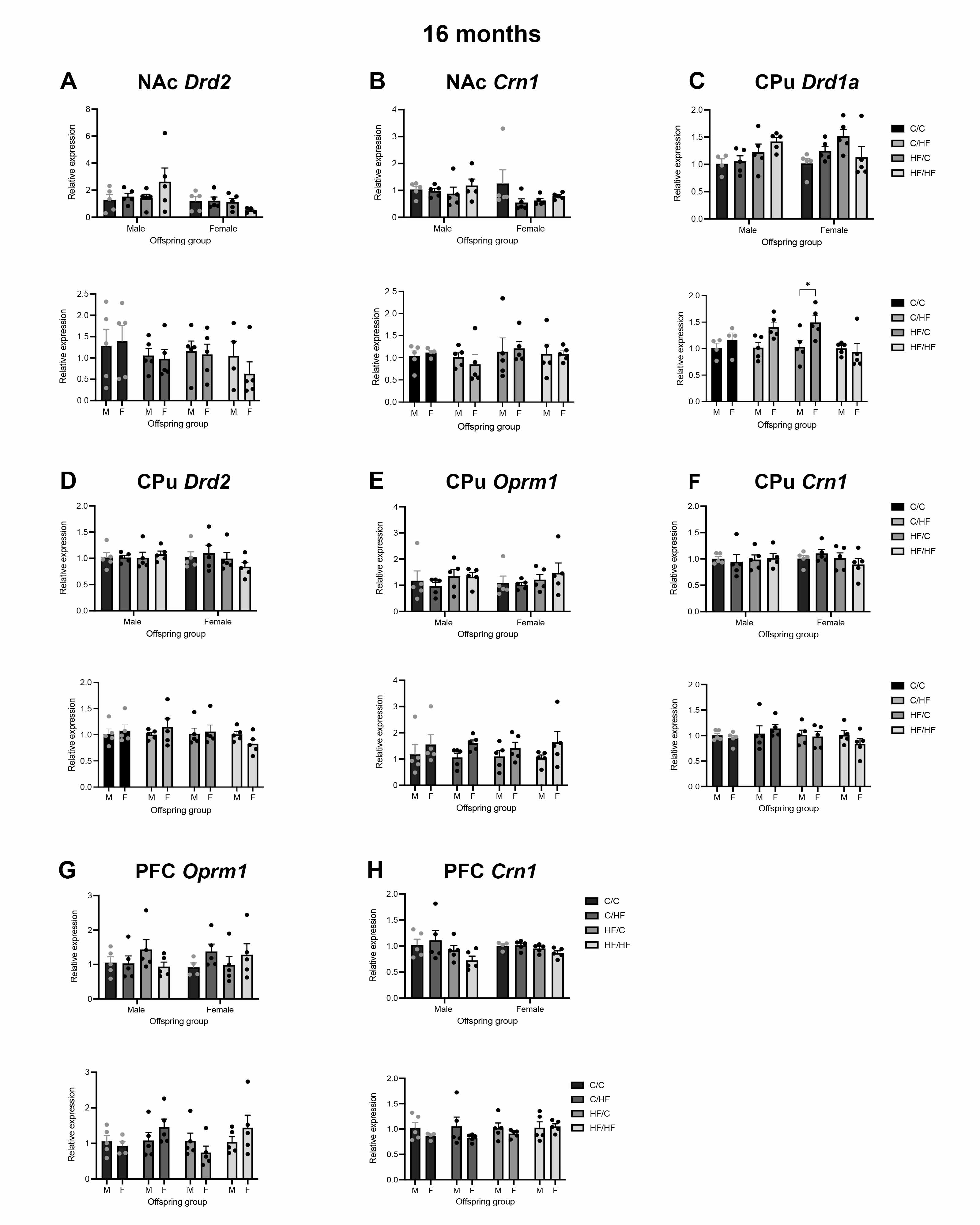

Supplement: Supplementary file 1 [file nutrients-14-05161-s001.zip › Supplemental Figure S5.jpg]

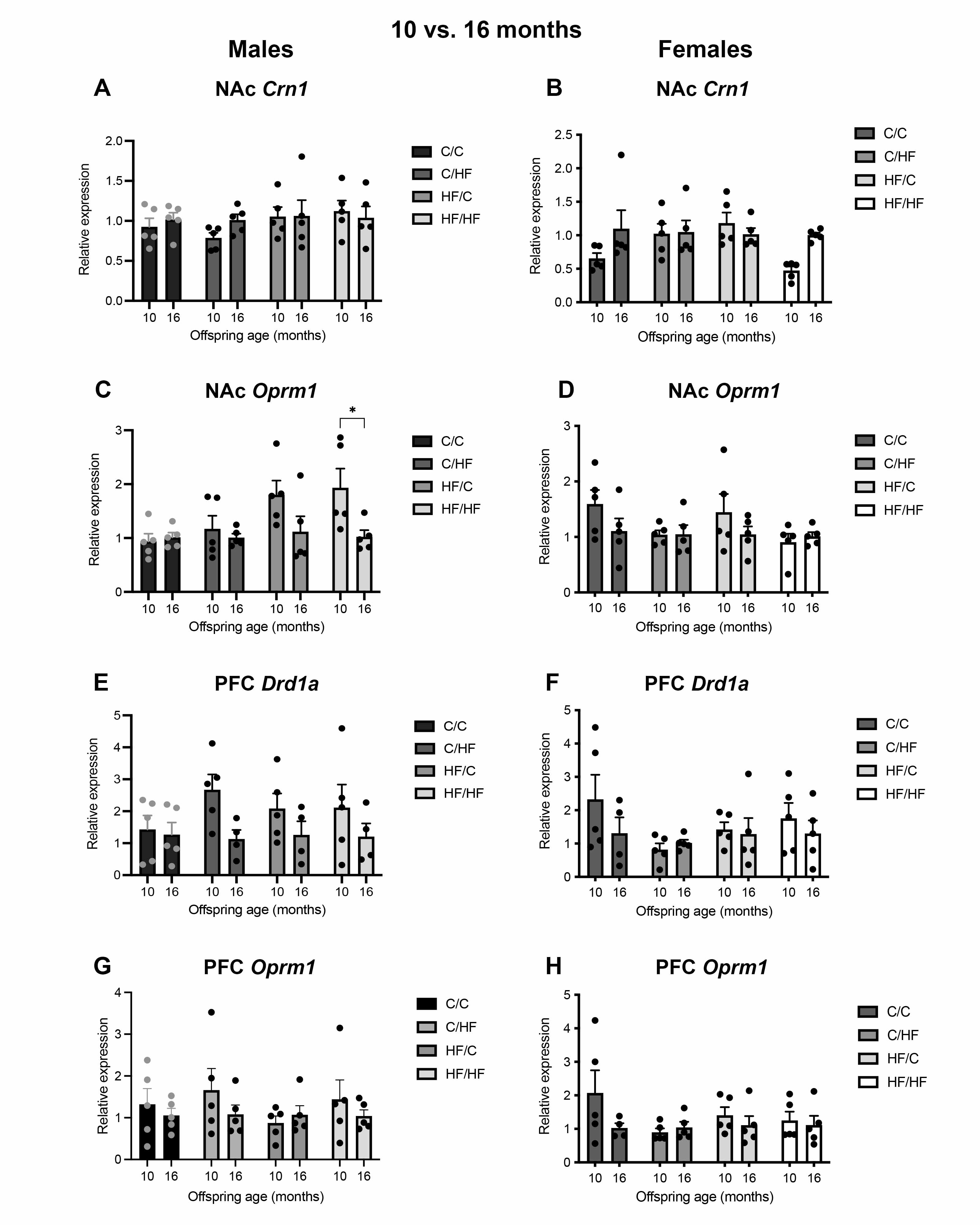

Supplement: Supplementary file 1 [file nutrients-14-05161-s001.zip › Supplemental Figure S6.jpg]
